# The Smc5–Smc6 Complex Is Required to Remove Chromosome Junctions in Meiosis

**DOI:** 10.1371/journal.pone.0020948

**Published:** 2011-06-22

**Authors:** Sarah Farmer, Pedro A. San-Segundo, Luís Aragón

**Affiliations:** 1 Cell Cycle Group, Medical Research Council Clinical Sciences Centre, Imperial College, London, United Kingdom; 2 Instituto de Biología Funcional y Genómica, Consejo Superior de Investigaciones Científicas / University of Salamanca, Salamanca, Spain; National Cancer Institute, United States of America

## Abstract

Meiosis, a specialized cell division with a single cycle of DNA replication round and two consecutive rounds of nuclear segregation, allows for the exchange of genetic material between parental chromosomes and the formation of haploid gametes. The structural maintenance of chromosome (SMC) proteins aid manipulation of chromosome structures inside cells. Eukaryotic SMC complexes include cohesin, condensin and the Smc5–Smc6 complex. Meiotic roles have been discovered for cohesin and condensin. However, although Smc5–Smc6 is known to be required for successful meiotic divisions, the meiotic functions of the complex are not well understood. Here we show that the Smc5–Smc6 complex localizes to specific chromosome regions during meiotic prophase I. We report that meiotic cells lacking Smc5–Smc6 undergo catastrophic meiotic divisions as a consequence of unresolved linkages between chromosomes. Surprisingly, meiotic segregation defects are not rescued by abrogation of Spo11-induced meiotic recombination, indicating that at least some chromosome linkages in *smc5–smc6* mutants originate from other cellular processes. These results demonstrate that, as in mitosis, Smc5-Smc6 is required to ensure proper chromosome segregation during meiosis by preventing aberrant recombination intermediates between homologous chromosomes.

## Introduction

Sexual organisms require a specialized cellular division, known as meiosis, to reduce their chromosome number by half to produce gametes [reviewed in [1], [2]. This process entails a division with two rounds of chromosome segregation and only one of DNA replication. The chromosome-halving event occurs in the first division, during which homologous chromosomes pair up and undergo recombination, generating crossovers (CO) between them. COs give rise to genetic variability but also, importantly, are crucial for chromosome segregation because they act as the physical linkages necessary for the correct orientation of homologues on the first meiotic spindle.

The number of COs and their position is an important issue; if a chromosome pair fails to establish COs, the homologues may not segregate to opposite poles and, similarly, if too many COs are established between homologues, timely separation may not take place [3]. The formation of COs, therefore, is necessarily a highly regulated process, promoted by factors such as the ZMM proteins [4], and antagonized by others, for example the Sgs1 helicase [5], [6], [7]. As a consequence, the number of DNA double-strand breaks (DSBs) initiating recombination far exceeds the number of COs [8].

Upon DSB induction by the nuclease Spo11 [9], [10], breaks are resected, and the protruding overhangs are able to invade homologous sequences in the sister and homologue duplexes. Although it was generally thought that recombination between homologue duplexes dominates during meiosis, recent evidence demonstrates that sister duplexes are also used extensively for the repair of meiotic DSBs [11]. During recombination, single-end invasions undergo regulation to drive the appropriate outcomes at different sites [12], including COs and non-COs [13]. Joint molecules (JM) are precursor intermediates for CO formation. The Sgs1 helicase and Mus81-Mms4 endonuclease suppress excessive JMs, including aberrant multichromatid JMs, as they cause segregation defects during the first division. [6], [14], [15].

Sgs1 has also been shown to dissolve inter-chromatid junctions originating during mitotic recombinational repair [16], [17]. Mitotic chromatid junctions, like meiotic joint molecules, are caused by defects in recombination and interfere with chromosome segregation [18]. Sister chromatid junctions accumulate in mutants of the Smc5–Smc6 complex [18], [19], [20], [21], a conserved multi-subunit complex involved in DNA repair via homologous recombination [22]. The complex consists of six non-SMC subunits, named Nse1-6, in addition to the Smc5 and Smc6 heterodimer [23], [24], [25]. Nse2, also known as Mms21, has SUMO ligase activity and promotes the sumoylation of several proteins [26], [27], [28]. Given its roles in mitotic recombination and some studies in fission yeast demonstrating that the Smc5–Smc6 complex is necessary during meiosis [29], the complex is anticipated to feature prominently in the metabolism of meiotic DSBs and recombination. However its exact function remains elusive.

## Results

### Smc5–Smc6 localization during prophase I is not dependent on the formation of Spo11-DSBs

**Figure 1:**
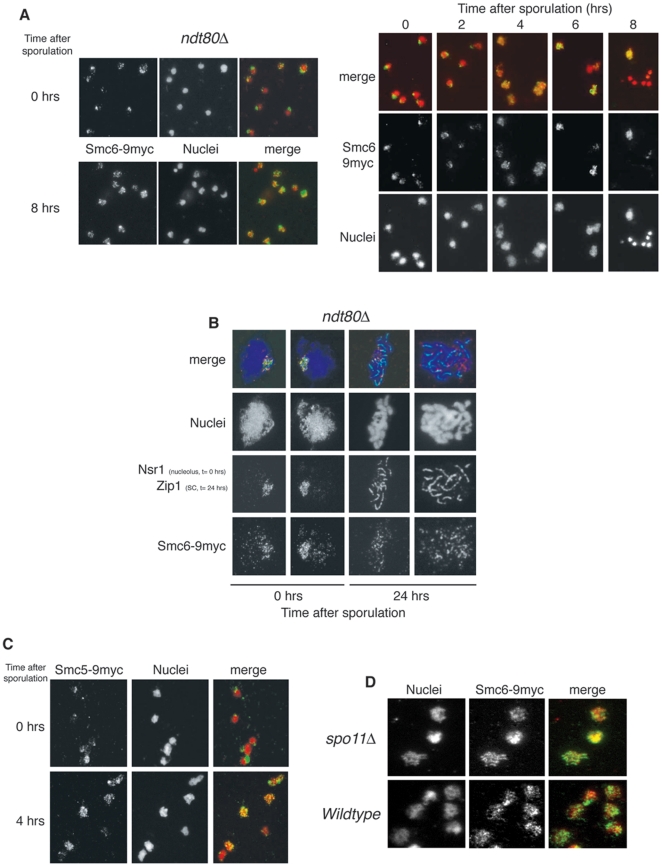
Smc5–Smc6 complex relocalises to specific regions during meiotic prophase I. (A) SK1 nuclei from *ndt80Δ* cells expressing *SMC6-9MYC* (CCG2422) harvested at different time points in meiosis (0 and 8 hrs) were surface spread and stained with anti-myc antibodies and DAPI (left panel). Synchronously sporulating SK1 nuclei from wildtype cells expressing *SMC6-9MYC* (CCG1508) harvested at the time points indicated were surface spread and stained with anti-myc antibodies and DAPI (right panel). (B) BR nuclei from *ndt80Δ* cells expressing *SMC6-9MYC* (CCG5019) harvested at different time points in meiosis (0 and 24 hrs) were surface spread and stained with anti-Zip1 (Synaptonemal Complex; 24 hrs), anti-Nsr1 (nucleolus; 0 hrs) and anti-myc antibodies. (C) Synchronously sporulating SK1 nuclei from wildtype cells expressing *SMC5-9MYC* (CCG1103) harvested at 0 and 4 hours of meiosis were surface spread and stained with anti-myc antibodies and DAPI. (D) SK1 nuclei from *spo11Δ* cells expressing *SMC6-9MYC* (CCG3830) harvested at 4 hrs after meiotic induction (prophase I) were surface spread and stained with anti-myc antibodies.

To begin to dissect the meiotic function of Smc5–Smc6, we first examined the localization of its subunits on chromosomes during synchronized meioses (Figure 1A). We used Smc6p as a representative of the complex. A COOH-terminal myc-tagged allele of *SMC6* was incorporated into the endogenous locus, providing the sole functional copy in the genome. Nuclei from meiotic cells expressing Smc6p-9myc were spread onto slides and processed for indirect immunofluorescence. In early meiosis, Smc6p-9myc is enriched in the nucleolus: a characteristic localization pattern also found in mitotic cells [18]. As cells progress into meiosis, Smc6p-9myc redistributes into distinct foci throughout the rest of the nucleus (Figure 1A). The appearance of non-nucleolar foci is maximal 4–6 hr after induction, corresponding to the prophase I period (Figure 1A; right panels). Smc6p-9myc localization in cells arrested in pachytene, by deletion of the meiotic transcription factor Ndt80 [30], confirms the punctate nuclear distribution (Figure 1A; left panels). Chromosome spreads of BR background *ndt80Δ* cells expressing *SMC6-9MYC* confirm the formation of foci and demonstrate their colocalization with meiotic chromosomes (Figure 1B). Smc5p-9myc exhibits a similar pattern (Figure 1C). This pattern of localization is similar to various markers of meiotic recombination [31], [32] which, added to the fact that the Smc5–Smc6 complex is recruited to mitotic DSBs [33], [34], [35], prompted us to test whether the punctuate nuclear distribution of Smc5–Smc6 is dependent on meiotic DSBs. Deletion of the *SPO11* gene prevents DSB formation but not sporulation and, in the absence of meiotic recombination, random segregation renders spores from *spo11Δ* cells largely inviable [36]. Smc6p-9myc forms chromosomal foci in *spo11Δ* cells (Figure 1D), demonstrating that Smc5-Smc6 relocalization to prophase chromosomes is DSB-independent.

### Meiotic nuclear divisions require Smc5–Smc6

**Figure 2:**
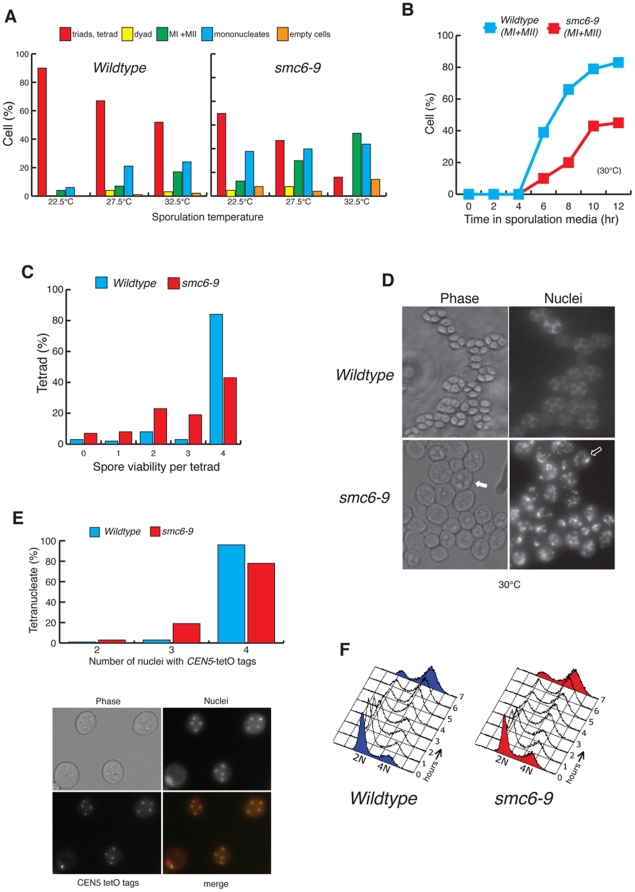
Meiotic catastrophe in *smc6–9* cells. (A) SK1 wildtype (CCG2009) and *smc6–9* (CCG1985) cultures scored after 3 days on solid sporulation media at the indicated temperatures for meiotic products. The analysis includes meiotic divisions (MI + MII) and sporulation (tetrads, triads and dyads) in the cultures. (B) Timing and efficiency of meiotic divisions in parallel cultures of SK1 wildtype and *smc6–9* strains at 30°C. MI + MII represents cells that have completed one or both meiotic divisions. (C) Spore viability, assessed by dissection of tetrads for SK1 wildtype and *smc6–9* strains after 24 hrs sporulation in liquid media at 30°C. (D) DAPI fluorescence and bright-field images of cells from wildtype and *smc6–9* cultures sampled 24 hrs after meiotic induction at 30°C. Arrows indicate immature asci: the filled arrow highlights an uncondensed ascus and the open arrow designates spores with immature spore walls through which DAPI bodies are visible.(E) Quantification of nuclei carrying GFP dots at *CEN5* for SK1 wildtype (CCG6864) and *smc6–9* (CCG6937) cells containing four nuclei only. Representative micrographs show *CEN5* GFP, DAPI fluorescence and bright-field images. (F) Flow cytometry analysis of SK1 wildtype (CCG2009) and *smc6–9* cultures (CCG1985) at the indicated times after meiotic induction at 30°C.

*SMC6*, like all identified subunits of the Smc5–Smc6 complex, is an essential gene. Temperature sensitive (ts) alleles of Smc6 have been employed to study the function of the complex in mitosis [18]. We followed the same approach in diploid cells and replaced both copies of *SMC6* with the conditional mutant allele *smc6–9*. The sporulation efficiency of *smc6–9* was compared to that of the wildtype at different temperatures, since meiosis is inherently temperature-sensitive. We found sporulation in *smc6–9* to be significantly lower than in wildtype cells at all temperatures (Figure 2A). Reduced sporulation frequencies were also found for other *smc5–smc6* ts alleles, including *smc5–6*, *nse3–12* and *nse5–1* (Figure S1). Despite the reduction in the quantitiy of spores produced (Figure 2A), analysis of kinetics of meiosis in synchronized *smc6–9* cultures showed that the onset of meiotic divisions was not significantly delayed in the mutant (Figure 2B). However the number of cells undergoing both divisions was considerably lower than that observed for wild-type cells (Figure 2B) thus explaining the reduced sporulation (Figure 2A).

We next examined whether the few tetrads formed in *smc6–9* (∼15% at 32.5°C) (Figure 2A), contain viable meiotic products. A reduced viability was observed for *smc6–9* tetrads, with only 45% containing four viable spores (Figure 2C). In addition, we suspect this spore viability to be an overestimate because many *smc6–9* tetrads, which appear immature (Figure 2D), are unlikely to survive zymolyase treatment pre-dissection.

The spore viability pattern (4, 2, 0 viable spores >3 and 1) is consistent with high levels of meiosis I nondisjunction (Figure 2C) and many *smc6–9* nuclei fail to divide at all (Figure 2A–B). Furthermore, some *smc6–9* cells undergo partial divisions with the appearance of fragmented nuclei (Figure 2D). This is indicative of a defect in the segregation of chromosomes. To evaluate this possibility in a direct manner, we scored the segregation of chromosome V marked with *tet* operator repeats 1.4 kb away from the centromere (*CEN5* dots). To simplify the analysis we scored only tetranucleated cells (Figure 2E). We found no defects in premeiotic pairing in *smc6–9* (data not shown). However, over 20% of *smc6–9* cells harbour nuclei lacking chromosome V (Figure 2E), compared to only 3% of wildtype cells, confirming a missegregation phenotype.

The large proportion of mononucleated cells in *smc6–9* mutants (Figure 2B) suggests a failure to enter meiosis or an arrest in prophase I. Flow-cytometry analysis of synchronized meiotic *smc6–9* cultures shows that the majority of cells enter meiosis and complete pre-meiotic replication (Figure 2F).

**Figure 3:**
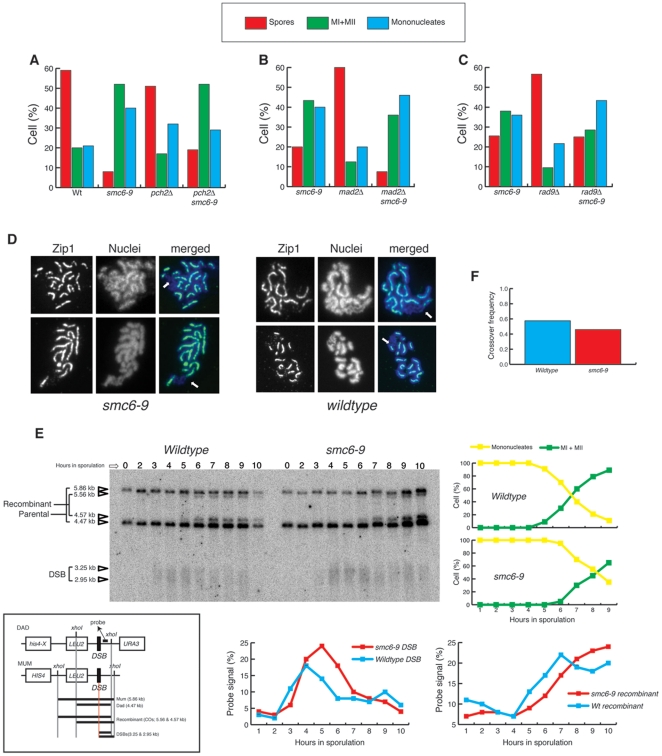
Meiotic recombination is unaffected in *smc6–9* cells. (A) Analysis of meiotic divisions (mononucleates, MI+MII) and sporulation (spores) in parallel cultures of SK1 wildtype (CCG2009), *smc6–9* (CCG1985), *pch2Δ* (CCG2425) and *pch2Δ smc6–9* (CCG2424), sporulating at 32.5°C on solid media. “Spores” indicates cells that contain at least two spores. (B) Analysis of meiotic divisions (mononucleates, MI+MII) and sporulation (spores) in parallel cultures of SK1 *smc6–9* (CCG1985), *mad2Δ* (CCG6842) and *mad2Δ smc6–9* (CCG6866), sporulating at 25°C on solid media. “Spores” indicates cells that contain at least two spores. (C) Analysis of meiotic divisions (mononucleates, MI+MII) and sporulation (spores) in parallel cultures of SK1 *smc6–9* (CCG1985), *rad9Δ* (CCG7182) and *rad9Δ smc6–9* (CCG7178), sporulating at 25°C on solid media. “Spores” indicates cells that contain at least two spores. (D) Pachytene BR nuclei from wildtype (BR1919) and *smc6–9* (CCG4874) cultures were surface spread and stained with anti-Zip1 (Synaptonemal Complex) and DAPI. Arrows indicate rDNA. (E) Physical analysis of recombination at the *HIS4-LEU2* locus at the indicated times after induction of sporulation at 30°C in SK1 wildtype (CCG3970) and *smc6–9* (CCG3976) cultures. Map of the *HIS4-LEU2* locus showing diagnostic restriction sites and position of the probe is shown. Image of a representative 1D Southern analysis, indicating DNA species, and quantitative analysis of DSBs, crossovers (recombinant), and meiotic divisions (MI +MII). “% probe signal” is percent of total hybridizing DNA per lane. (F) Analysis of meiotic recombination frequency at the *HIS4-LEU2* locus between *HIS4* and *URA3* in SK1 wildtype and *smc6–9* tetrads after 24 hrs sporulation at 30°C.

The pachytene checkpoint [37], dependent on Pch2p [38], acts to prevent the first nuclear division when meiotic recombination or synapsis is incomplete. We therefore tested whether *pch2Δ* *smc6–9* meiotic cultures contain mononucleated cells. Although a small reduction in mononucleated cells was observed in *pch2Δ* *smc6–9* compared to *smc6–9* (Figure 3A), the majority of mononucleated cells in *smc6–9* are not arrested by the Pch2-dependent checkpoint.

The independence of *smc6–9* from the Pch2-dependent checkpoint prompted us to investigate whether the spindle checkpoint, known to be functioning during the first meiotic division [39], is activated in these cells. Deletion of *MAD2* in *smc6–9*, however, did not reduce the number of mononucleates (Figure 3B), hence activation of the spindle checkpoint is not responsible for the accumulation of this cell type. Similarly, no mononucleate suppression in *smc6–9* meioses was observed when the DNA damage checkpoint adaptor *RAD9* was deleted (Figure 3C), demonstrating that meiotic DNA damage checkpoints [40] are not activated in *smc6–9* mutant meioses.

### Defective meiotic recombination is not the sole cause of chromosome segregation defects in *smc6*–*9* meioses

Analysis of meiotic progression in *smc6–9* mutant cells revealed a defect during nuclear division that manifests as severe chromosome missegregation (Figure 2D–E). In vegetative cells, *smc6–9* lethality is suppressed when homologous recombination is blocked [18]. To investigate the interplay between Smc5–Smc6 and recombination in meiosis, we first investigated whether *smc6–9* mutants are able to synapse homologous chromosomes normally. Mutants that fail to undergo effective meiotic recombination often show a redistribution of the Synaptonemal Complex (SC) protein Zip1p into polycomplexes during pachytene [41]. We probed wildtype and *smc6–9* pachytene-arrested nuclei (by *ndt80Δ*) with an antibody raised against Zip1p. The pattern of Zip1p distribution is similar in wildtype and *smc6–9* spreads (Figure 3D); binding is linear and confined to the central region of pachytene chromosomes, moreover Zip1p is absent from the rDNA (Figure 3D; arrows).

To determine at the molecular level whether the meiotic catastrophe in *smc6–9* is caused by meiotic recombination defects at the molecular level, we monitored the DNA events of meiotic recombination using the *HIS4-LEU2* physical assay system [9], [42], [43]. Wildtype and *smc6–9* mutant cells were induced to sporulate synchronously and samples collected at hourly intervals for assessment of the recombination status of the *HIS4-LEU2* locus (Figure 3E). In wildtype cells, DSBs are first detected 3 hr after transfer to sporulation medium and disappear by 6 hr (Figure 3E). In *smc6–9* cells, DSB dynamics are similar but delayed by approximately an hour (present between 4 and 7 hrs) (Figure 3E). In both wildtype and *smc6–9* cells, crossover levels rise as the amount of DSBs decline (Figure 3E), indicating that *smc6–9* cells are able to process meiotic DSBs into recombinant products. Furthermore, recombination at the locus (between *HIS4* and *URA3*), measured genetically in the 4 spore-viable tetrads, revealed no significant differences between wildtype (Figure 3F; with a frequency of 0.6) and *smc6*–*9* (Figure 3F; with a frequency of 0.47). In addition, we measured crossing over in four intervals on chromosome XV [44] by tetrad analysis. In all intervals, crossing over in the *smc6–9* mutant is comparable to wildtype (Fig. S2), however, we found an increase in gene conversion events (Fig. S2) demonstrating that recombination is upregulated in *smc6–9* cells.

**Figure 4:**
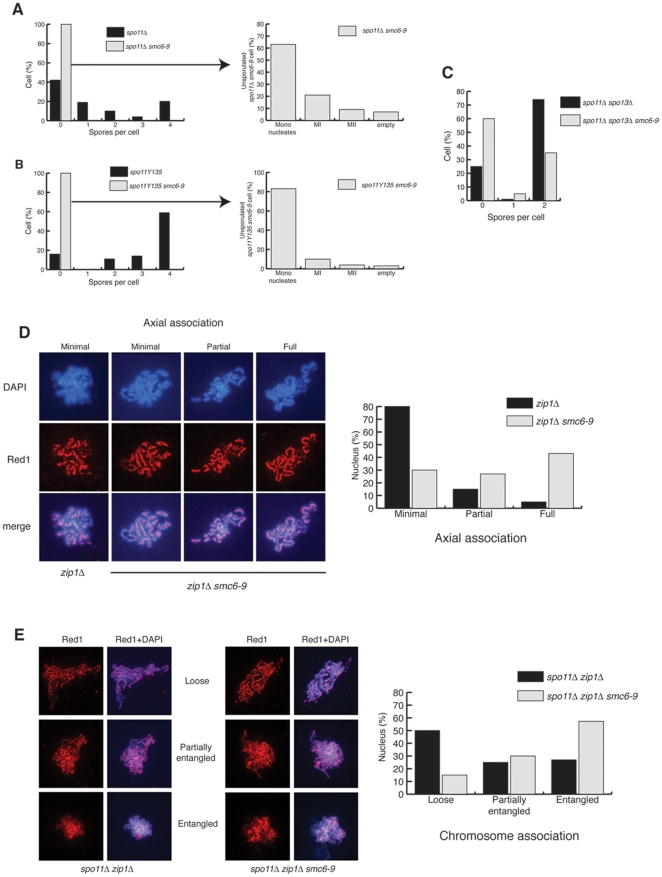
*spo11Δ* does not rescue *smc6–9* despite pseudosynapsis in *zip1Δ smc6–9* mutants. (A) Analysis of sporulation efficiency (left graph) and meiotic divisions in unsporulated cells (right graph) in SK1 *spo11Δ* (CCG2396) and *spo11Δ smc6–9* (CCG2429), sporulated at 25°C for 3 days on solid media. (B) Analysis of sporulation efficiency (left graph) and meiotic divisions in unsporulated cells (right graph) in SK1 *spo11-Y135* (CCG3733) and *spo11-Y135 smc6–9* (CCG4498), sporulated at 25°C for 3 days on solid media. (C) Analysis of sporulation efficiency in SK1 *spo11Δ spo13Δ* (CCG4678) and *spo11Δ spo13Δ smc6–9* (CCG4680), sporulated at 25°C for 3 days on solid media. (D) BR pachytene-arrested *ndt80Δ zip1Δ* (DP428) and *ndt80Δ zip1Δ smc6–9* (DP687) nuclei were surface spread and stained with anti-Red1 (chromosome cores) and DAPI. Representative micrographs for the different categories of axial association are shown. Quantification of axial association is shown (graph). The number of nuclei scored was 119 for *ndt80Δ zip1Δ* and 122 for *ndt80Δ zip1Δ smc6–9*. (E) BR pachytene-arrested *ndt80Δ spo11Δ zip1Δ* (DP728) and *ndt80Δ spo11Δ zip1Δ smc6–9* (DP727) nuclei were surface spread and stained with anti-Red1 (chromosome cores) and DAPI. Representative micrographs for the different categories of chromosome association are shown. Quantification of chromosome association is shown (graph). The number of nuclei scored was 100 for *ndt80Δ spo11Δ zip1Δ* and 102 for *ndt80Δ spo11Δ zip1Δ smc6–9*.

*sgs1Δ* and *mms4Δ* mutants incur catastrophic divisions during meiosis [7], [45], [46], [47], [48], [49], [50]. Previous analyses of meiotic Sgs1p and Mms4p depletion [6], [14], [15] revealed no changes in recombination at the *HIS4-LEU2* hotspot, yet showed suppression of nuclear division defects when meiotic Spo11-dependent DSBs were abolished [14], [15]. Similarities in the phenotypes of *sgs1Δ* and *smc6–9*, both in mitosis and meiosis, together with the increase in gene conversions (Fig. S2), prompted us to analyse the effect of DSB abrogation on spore viability in *smc6–9*. Meiotic DSBs were precluded by deletion of *SPO11* or by its replacement with a catalytically inactive allele, *spo11-Y135F* [51] (Figure 4A–B). Strikingly, inactivation of Spo11 function does not suppress the catastrophic meiosis of *smc6–9* but, in fact, further decreases the efficiency of nuclear divisions and ascus formation (Figure 4A–B compared to Figure 2A). Similar results were obtained for other *smc5–* *smc6* ts alleles, including *smc5–* *6*, *nse3–* *12* and *nse2Δ* *C* (data not shown). These results suggest that the chromosome segregation defects in *smc6–9* cells are largely caused by defects unrelated to Spo11p-induced recombination. Deletion of *SPO13*, which enables viable spore production in a *spo11Δ* background via a wholly equational division [52], [53], permits some spore formation in *spo11 smc6–9* (Figure 4C compared to 4A). However, the simultaneous inactivation of Spo11 and Spo13 fails to fully rescue sporulation in *smc6–9* (Figure 4C). Together, these observations imply that the Spo11-independent problems in *smc6–9* cells affect the segregation of both homologous chromosomes in the first meiotic division and sister chromatids in the second.

### *smc6–* *9* cells undergo pseudosynapsis in the absence of Zip1

The mitotic phenotype of haploid *smc6–9* cells is sister-chromatid nondisjunction during division, caused by unresolved recombination and incomplete replication [18], [19]. Diploid cells with compromised Smc5–Smc6 function exhibit a 100-fold increase in loss of heterozygosity [54], indicating that there is a significant increase in recombination between homologous chromosomes in mitosis in the absence of Smc5–Smc6. Our results demonstrate that the *smc6–9* mutant undergoes a catastrophic meiosis where segregation in both meiotic divisions is affected (Figure 2A, C, D, E & 4C. Surprisingly, abolition of meiotic recombination does not suppress this phenotype (Figure 4A, B & C), suggesting that the meiotic segregation defects might be a result of unresolved recombination between homologues that originates, not only from programmed meiotic DSBs, but also from lesions caused by the lack of Smc6 function during premeiotic S phase. Furthermore, the increase in gene conversion (Fig. S2) suggests that increased recombination (Spo11-dependent or -independent) might be the cause of the *smc6–9* catastrophic divisions. To address this possibility, we investigated whether the *smc6–9* mutant exhibits an increase in connections between homologous chromosomes during prophase I.

In wildtype cells, the absence of SC protein Zip1p leads to linkage of homologue axes only at sites of crossing over, which are clearly visible in nuclear spreads of pachytene cells [7] (Figure 4D; 1^st^ panel). Upregulation of recombination between homologues, as in *sgs1Δ zip1Δ* mutants, restores close association between homologue axes, which is referred to as pseudosynapsis [7]. To test whether homologues in *smc6–9* are excessively linked, we deleted *ZIP1*. We stained Red1p, a component of the chromosome core [55], to evaluate homologue connections in pachytene (by *ndt80Δ* arrest). As expected, in the *zip1Δ* single mutant, individual chromosome cores are joined only by periodic axial associations representing sites of crossing over [7] (Figure 4D). In contrast, most *zip1Δ smc6–9* spreads appear fully synapsed (Figure 4D). We conclude that, in the absence of Smc6p function, the presence of excessive homologue linkages causes catastrophic segregation. To further investigate whether chromosomal junctions are present in the absence of Spo11-dependent DSBs, we compared chromosomal associations in nuclear spreads of *zip1Δ spo11Δ* and *zip1Δ spo11Δ smc6–9* cells (Figure 4E) arrested in pachytene (by *ndt80Δ*). Red1p staining in *zip1Δ spo11Δ* spreads shows that association between homologue axes is absent, and individualized chromosomes are observed in the majority of cells (Figure 4E; Loose category). In *zip1Δ spo11Δ smc6–9* spreads, however, despite the fact that we do not find pseudosynapsis, individualised chromosomes are not observed (Figure 4E; Loose category). Instead, most nuclei present as an entangled mass of chromosomes (Figure 4E; Entangled category). This result is consistent with our previous demonstration that deletion of *SPO11* does not rescue *smc6–9* defects (Figure 4A–C), and suggests that the presence of Spo11-independent junctions between chromosomes contributes to the meiotic segregation defects observed in *smc6–9* mutants.

## Discussion

Here we have characterized the meiotic phenotypes of several *smc5–smc6* mutants during budding yeast meiosis. Our results demonstrate that cells lacking functional Smc5–Smc6 undergo highly aberrant divisions where chromosomes fail to segregate correctly due to excessive and unresolved linkages between chromosomes during prophase I. Surprisingly, these defects are not dependent on meiotic recombination, as *smc5–smc6 spo11Δ* mutants are also affected. We therefore propose that the function of Smc5–Smc6 is crucial during premeiotic S phase, where the complex acts to prevent or correct excessive linkages between chromosomes that interfere with chromosome segregation in the first meiotic division.

## Methods

### Strains and Growth conditions

All yeast strains used in this study were from SK1 or BR genetic backgrounds and are shown in Table S1. The genetic background of the strains used for each experiment is indicated in each figure legend. For synchronous meioses (SK1 genetic background), freshly-streaked large isolated colonies were cultured individually to saturation in 5 to 10 ml YPD. These cultures were used to inoculate 50 to 200 ml YP potassium acetate (1%) at pH 5.5 in 10x-volume conical flasks to 0.2 OD595. Flasks were shaken at 25°C or 30°C for 12 to 18 hours at maximum speed and cultures measuring 1.1<OD595<1.5 and comprising >80% large, unbudded cells were selected to be meiotically induced. These were rapidly washed twice in half-volumes of distilled water pre-equilibrated to 30°C and were resuspended in equal-volumes of sporulation medium pre-equilibrated to, and subsequently incubated at, 30°C or the appropriate experimental temperature. Sporulation media was either 0.3% or 1% potassium acetate with 0.02% raffinose or 2% potassium acetate, depending on the lab of origin of the strains used. Cultures were shaken at maximum speed in 10x-volume conical flasks.

For meiotic induction of BR genetic background, isolated colonies were grown in a third-volume of the eventual desired culture volume of 2x synthetic complete media supplemented with 1 g/l adenine at 30°C for 20 hours, or at 25°C for 24 hours where thermosensitive strains were used. Cells were then resuspended in a quarter-volume of the eventual desired culture volume of YPDA supplemented with 400 µM adenine and 200 µM uracil and were grown for 8 further hours at the same temperature. After 1 wash in 2% potassium acetate, cells were finally resuspended in 2% potassium acetate to induce meiosis at 30°C.

For sporulation on solid media, isolated colonies were grown to saturation in 5 ml YPD, washed twice in distilled water and resuspended in 1 ml distilled water. Patches, covering approximately one eighth of a standard petri dish, were made by pipetting 100 µl cell suspension onto sporulation agar supplemented with 1/4x complete supplement mixture. Plates were incubated for 3 days.

For tetrad dissection, sporulated cells were resuspended in distilled water with 0.05 mg/ml 100 T zymolyase and incubated at room temperature for 10 minutes before plating.

### Flow cytometry

For analysis by flow cytometry, cells were fixed in 70% ethanol for 1 hour at room temperature, resuspended in SSC plus 0.1 mg/ml RNase A and incubated at 50°C overnight. Proteinase K was added to a final concentration of 0.1 mg/ml and cells were incubated for a further hour at 50°C. Finally, a five-third volume of 5 µg/ml propidium iodide in SSC was added and cells were incubated in the dark at room temperature for 1 hour. Flow cytometric analysis was performed on a FACScan cytometer (Becton Dickinson) using CellQuest Pro (Becton Dickinson) software.

### Cytology

For SK1 nuclear spreads, cells were washed in ice-cold KS buffer (1.2 M sorbitol, 2% potassium acetate), resuspended in ice-cold KS buffer with 0.01 M DTT and 100 µg/ml 100 T zymolyase and incubated at 37°C for 20 minutes or until 95% of the cells were spheroplasted (lysed in 1% SDS). Spheroplasts were gently washed in ice-cold MS buffer (1.2 M sorbitol, 0.1 M MOPS, 1 mM EDTA, 0.5 mM magnesium chloride, 1 mM PMSF) and resuspended in 20 µl per slide MS buffer. For spreading, 20 µl cell suspension was pipetted onto an acid-washed, ethanol-rinsed glass slide, rapidly followed sequentially by 80 µl fixative (4% paraformaldehyde, 4 mM potassium hydroxide, 10 mM MOPS), 40 µl 1% PhotoFlo and 80 µl fixative. A pipette tip was used to gently smooth the mixture over the surface of the slide before air-drying.

For immunostaining of SK1 nuclear spreads, slides were washed for 10 minutes in PBS and blocked for 10 minutes in blocking buffer (5% w/v BSA, 2% skimmed powdered milk in PBS) in a humidity chamber, before a 1-hour incubation at room temperature with mouse monoclonal anti-c-myc IgG1κ antibody 9E10 (1/1000; Roche) in blocking buffer. After a PBS wash, slides were incubated in the dark in FITC-conjugated goat anti-mouse (1/1000; Abcam) in blocking buffer for 1 hour. Following several PBS washes in the dark, 0.5 µl 0.1 µg/ml DAPI in mounting medium with Antifade was added to the slide before mounting with a coverslip.

Nuclear spreads for BR strains were prepared according to a modified version of the method described by Dresser and Giroux. Cells from 7 ml culture (per 3 slides) were collected in a round-bottomed tube, resuspended in 1 ml spheroplasting solution (2% potassium acetate, 1 M sorbitol, adjusted to pH 7, 10 mM DTT, 0.5 mg/ml 20 T zymolyase, glusulase to a final dilution of 1/200) and incubated shaking gently at 30°C for 20 minutes or more if required. Spheroplasted cells (95% lysed in 1% sarcosyl), were centrifugated at low speed and the cell pellet was drained and washed gently in 1 ml ice-cold MESSORB (0.1 M MES, 1 M sorbitol, 1 mM EDTA, 0.5 mM magnesium chloride, adjusted to pH 6.4). The pellet was then resuspended by pipetting 200 µl ice-cold MES buffer (0.1 M MES, 1 mM EDTA, 0.5 mM magnesium chloride, adjusted to pH 6.4) onto the wall of the tilted tube and adding 720 µl 4% paraformaldehyde to “push” the mixture down to the pellet at the bottom of the tube, before swirling gently. The suspension was poured onto 3 slides, covered with a large coverslip and left for 30 minutes. The coverslip was then discarded and the slide rinsed gently in 2 ml 0.4% PhotoFlo and air-dried.

To immunostain BR nuclear spreads, slides were washed in PBS for 3 minutes with gentle agitation and blocked in 200 µl fetal bovine serum under a coverslip for 1 hour at room temperature in a humidity chamber. Coverslips were displaced and slides drained for 1 minute before incubation with the appropriate primary antibody or antibodies (mouse monoclonal anti-c-myc IgG1κ antibody 9E10 (1/333; Roche), rabbit anti-Zip1 (1/200; a gift from Shirleen Roeder), goat anti-c-myc (1/333; Abcam), rabbit anti-Red1 (1/200; a gift from Shirleen Roeder) or mouse anti-Nsr1 2.3b (1/20; a gift from Michael Snyder)) in 75 µl 3% BSA in PBS overnight at 4°C. Slides were washed 3 times in PBS for 5 minutes and incubated with the appropriate secondary antibody or antibodies (FITC-conjugated donkey anti-rabbit (1/200; Jackson ImmunoResearch), Alexa Flour® 594-conjugated goat anti-mouse (1/200; Molecular Probes, Invitrogen), FITC-conjugated goat anti-mouse, FITC-conjugated donkey anti-mouse or Cy3- conjugated donkey anti-goat (all three 1/333; Abcam)) in 75 µl 3% BSA in PBS for 1 hour at room temperature. Following 3 5-minute washes in PBS, slides were drained for 1 minute and DAPI/Antifade was added before mounting.

For fluorescence microscopy, series of z-focal plane images were collected on a Leica IRB using a Hamamatsu D742-95 digital camera and OpenLab™ software (Improvision). A tuneable light source (Polychrome IV (Photonics)) with a Xenon lamp or an ultraviolet mercury lamp (Leica) were used. Images in different z-axis planes were flattened into a two-dimensional projection and processed in OpenLab. To visualise the nuclei of intact cells, cells were resuspended in a final concentration of 1% Triton® X-100 and 25 ng/ml DAPI/Antifade.

### DNA Physical assays

Extraction of DNA from synchronously sporulating cells was performed according to a protocol based on the method described by Cao et al. [9]. Approximately 22 OD595 cells, fixed in 70% ethanol at −20°C, were washed twice with spheroplasting buffer (1 M sorbitol, 10 mM sodium phosphate buffer at pH 7), 50 mM EDTA) and were incubated for 30 minutes at 37°C in 500 µl spheroplasting buffer with 0.006% β-mercaptoethanol and 10 µg/ml 100 T zymolyase. Spheroplasts (95% cells disrupted in 1% SDS), were centrifugated at 4000 rpm for 3 minutes and incubated in 500 µl lyse solution (50 mM EDTA, 0.3% SDS, 200 µg/ml proteinase K) at 65°C for 30 minutes. After cooling on ice, 200 µl 5 M potassium acetate was mixed in by inversion and the suspension was incubated on ice for 20 minutes. Cell debris was removed by centrifugation and the supernatant was phenol chloroform extracted with an equal-volume of phenol chloroform 3 times, and chloroform extracted once, in the same way, rocking for 30 minutes to mix rather than vortexing. DNA was ethanol precipitated at −20°C for at least 1 hour by adding a tenth-volume of 3 M sodium acetate at pH 5.2, then a double-volume of ethanol, and was finally resuspended in 40 µl 10 mM Tris pH 8. DNA concentration was measured and 40 mg was digested per sample with *XhoI* overnight in a 35 µl reaction volume. Digested DNA was electrophoresed for 24 hours at 70 V in a 6% agarose gel in 1x TBE with an electrode distance of 30 cm.

Gels were stained in 0.25 µg/ml ethidium bromide in TBE for 1 hour and were visualised in a Biorad Gel Doc 2000 using Quantity One(R) software (Biorad). Gels were prepared for Southern blot by a 10-minute incubation with agitation in 0.25 M HCl and at least a 30-minute incubation with agitation in 0.4 M sodium hydroxide. DNA was transferred onto positively-charged nylon transfer membrane (Hybond-N+, Amersham Biosciences) by capillary action in 0.4 M sodium hydroxide for at least 24 hours. The blot was UV-crosslinked by the autocrosslinking function of the UV Stratalinker® 2400 (Stratagene) and was washed in 2x SSC before air-drying. To make the radiolabelled probe, a DNA fragment amplified from wildtype genomic DNA, using primers 5′-CTCGTTGGTGTGTAAATACG and 5′-GCAAGCACAATTCCGGCAA, was gel purified using the QIAquick Gel Extraction Kit (Qiagen) and labelled with 32P by employing the Megaprime DNA labelling system (GE Healthcare) using dCT32P. The probe was purified with a Sephadex™ G-50 DNA Grade column (NICK™ Column, Amersham Biosciences), boiled for 5 minutes and quenched on ice. The probe was hybridised to the blot, which was prehybridised in Church buffer without BSA (7% SDS, 1 mM EDTA, 0.25 M sodium phosphate buffer at pH 7.2) at 65°C for at least 2 hours, in 20 ml Church buffer without BSA at 65°C overnight. The blot was washed at 65°C several times in each of 3 increasingly stringent wash solutions: 2x SSC with 0.5% SDS, 1x SSC with 0.1% SDS and finally 0.1x SSC with 0.1% SDS, and was then exposed to a Phosphor Screen (Amersham Biosciences) at least overnight. The screen was scanned on a Storm 820 phosphorimager (Molecular Dynamics) using Storm scanner control (Molecular Dynamics) software and bands were quantified using ImageQuant(R) 5.2 (Molecular Dynamics) software.

## Supporting Information

Figure S1 **Reduced sporulation efficiency in various *smc5–smc6* mutants.** Analysis of sporulation efficiency in SK1 wildtype (CCG2009), *smc6–9* (CCG1985), *smc5–6* (CCG1981), *nse2ΔC* (CCG3818), *nse3–12* (CCG2407) and *nse5–1* (CCG2132) strains sporulated at 25°C on solid media.
(TIF)

Figure S2 **Analysis of recombination frequency in wildtype and *smc6–9* strains.** Schematic of the genetic assay described in [44] and shown in tables (top panel). Parallel cultures of wildtype (CCG6844) and smc6–9 (CCG6585) were sporulated at 25°C on solid media for 3 days. Tetrads were dissected and spore clones genotyped using auxotrophic markers for analysis of recombination in four consecutive genetic intervals on chromosome XV. Spore viability data for the 100 wildtype and 241 *smc6–9* tetrads dissected are summarised in the upper panel with the percentage of tetrads in each spore viability category detailed. Recombination frequencies are shown on the left hand side of the lower panel. Due to low numbers of 4 spore-viable tetrads in *smc6–9*, recombination frequencies for the indicated intervals reflect the pooled individual spore data from all tetrads, regardless of their spore viabilities. Rf refers to the recombination frequency in single spores, determined as recombinant/(parental+recombinant), and Rf x 100 values are comparable to the conventional measurement of genetic recombination in centiMorgans (cM), which is calculated from 4 spore-viable tetrads. The mean number of crossovers in the whole *URA3-HIS3* interval per spore is also shown (CO/spore). Gene conversion events in 4 spore-viable tetrads obtained for wildtype (n = 77) and *smc6–9* (n = 84) are shown in the lower right hand panel. In *smc6–9*, the gene conversions shown represent 1 triple gene conversion event, 1 double gene conversion event and 8 single gene conversion events. Analysis of crossover interference in wildtype and *smc6–9* strains.Crossover interference, refers to the phenomenon whereby the presence of a crossover decreases the probability that a crossover will form in adjacent regions. A crossover interference value of 1 indicates that there is no interference. Due to low numbers of 4 spore-viable tetrads in *smc6–9*, crossover interference was calculated from individual spore data from all tetrads, regardless of their spore viabilities, rather than from the conventional non-parental ditype observed/expected ratio, which is measured in 4 spore-viable tetrads. COC refers to coefficients of coincidence and is the ratio of the observed number of double crossovers in adjacent genetic intervals to the predicted number of double crossovers based on the Rf values. This is calculated by the formula COC = number of double crossovers in 2 adjacent intervals A and B/(Rf for interval A x Rf for interval B).
(TIF)

Table S1 **Yeast strains used in this study.**
(DOC)
