# Supplementary figures and images for: The Smc5–Smc6 Complex Is Required to Remove Chromosome Junctions in Meiosis
Source: PLoS One. 2011 Jun 22;6(6):e20948. doi: 10.1371/journal.pone.0020948 (PMC3120815; doi:10.1371/journal.pone.0020948)

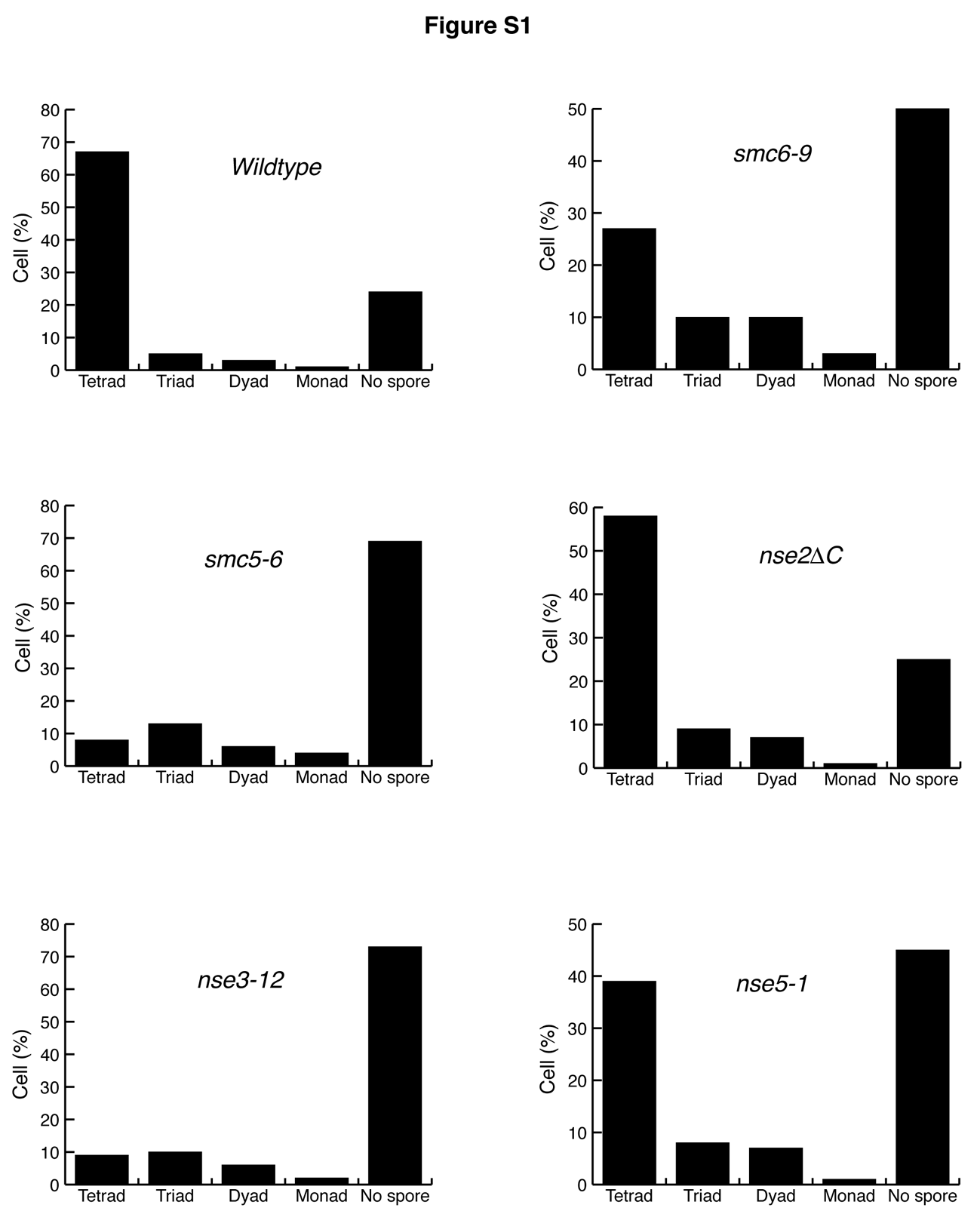

Supplement: Figure S1 — Reduced sporulation efficiency in various smc5–smc6 mutants. Analysis of sporulation efficiency in SK1 wildtype (CCG2009), smc6–9 (CCG1985), smc5–6 (CCG1981), nse2ΔC (CCG3818), nse3–12 (CCG2407) and nse5–1 (CCG2132) strains sporulated at 25°C on solid media. (TIF) [file pone.0020948.s001.tif]

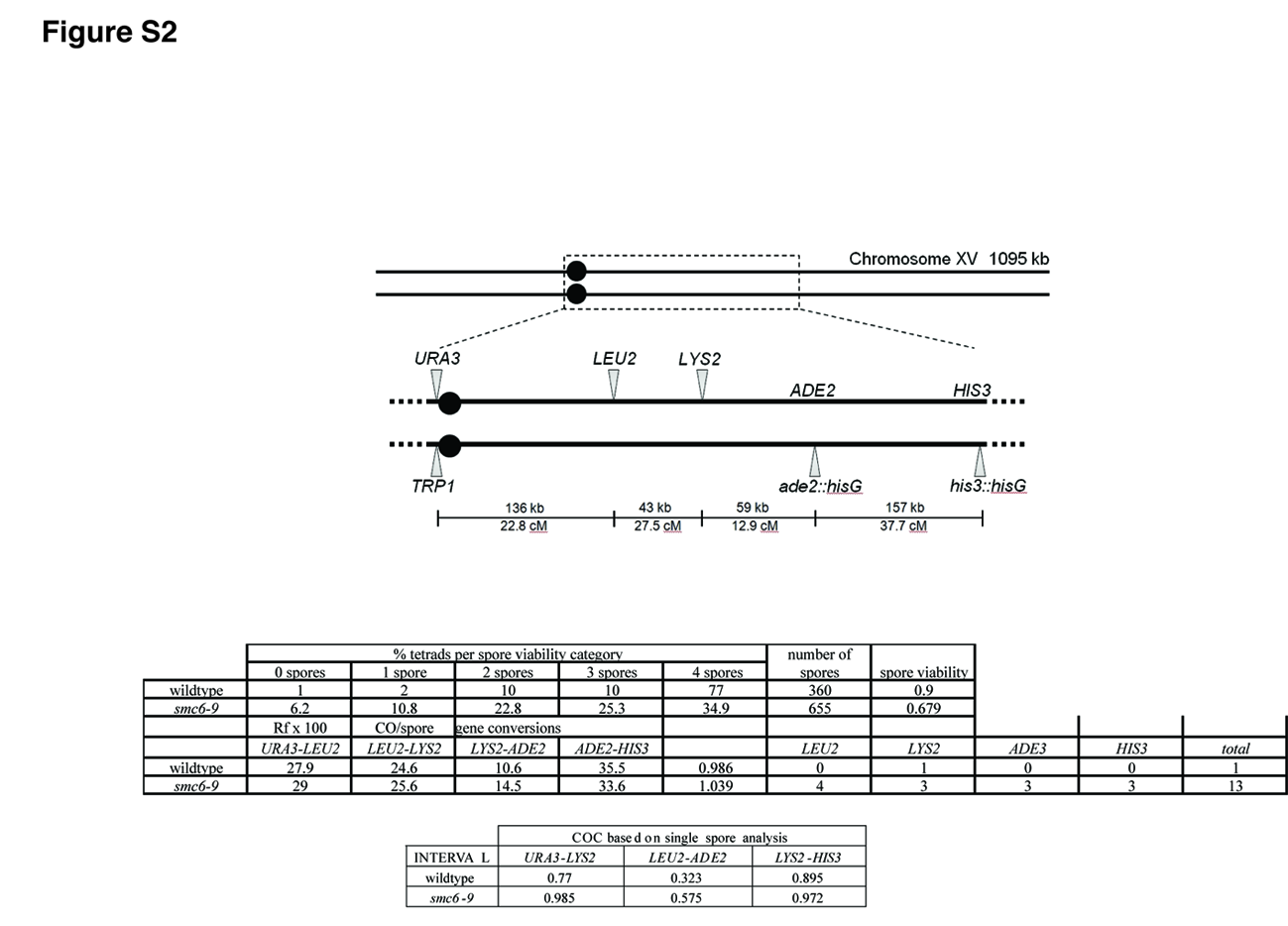

Supplement: Figure S2 — Analysis of recombination frequency in wildtype and smc6–9 strains. Schematic of the genetic assay described in [44] and shown in tables (top panel). Parallel cultures of wildtype (CCG6844) and smc6–9 (CCG6585) were sporulated at 25°C on solid media for 3 days. Tetrads were dissected and spore clones genotyped using auxotrophic markers for analysis of recombination in four consecutive genetic intervals on chromosome XV. Spore viability data for the 100 wildtype and 241 smc6–9 tetrads dissected are summarised in the upper panel with the percentage of tetrads in each spore viability category detailed. Recombination frequencies are shown on the left hand side of the lower panel. Due to low numbers of 4 spore-viable tetrads in smc6–9, recombination frequencies for the indicated intervals reflect the pooled individual spore data from all tetrads, regardless of their spore viabilities. Rf refers to the recombination frequency in single spores, determined as recombinant/(parental+recombinant), and Rf x 100 values are comparable to the conventional measurement of genetic recombination in centiMorgans (cM), which is calculated from 4 spore-viable tetrads. The mean number of crossovers in the whole URA3-HIS3 interval per spore is also shown (CO/spore). Gene conversion events in 4 spore-viable tetrads obtained for wildtype (n = 77) and smc6–9 (n = 84) are shown in the lower right hand panel. In smc6–9, the gene conversions shown represent 1 triple gene conversion event, 1 double gene conversion event and 8 single gene conversion events. Analysis of crossover interference in wildtype and smc6–9 strains.Crossover interference, refers to the phenomenon whereby the presence of a crossover decreases the probability that a crossover will form in adjacent regions. A crossover interference value of 1 indicates that there is no interference. Due to low numbers of 4 spore-viable tetrads in smc6–9, crossover interference was calculated from individual spore data from all tetrads, regardl [file pone.0020948.s002.tif]
