# Supplementary material for: The Smc5–Smc6 Complex Is Required to Remove Chromosome Junctions in Meiosis
Source: PLoS One. 2011 Jun 22;6(6):e20948. doi: 10.1371/journal.pone.0020948 (PMC3120815; doi:10.1371/journal.pone.0020948)
Supplement: Table S1 — Yeast strains used in this study. (DOC) [file pone.0020948.s003.doc]

**Supplemental information**

**Table S1**. Yeast strains used

| **Strain** | **Relevant genotype** | | **Reference** |
| --- | --- | --- | --- |
| CCG1103 | MATa/α leu2/leu2 ura3/ura3 lys2/lys2 trp1/trp1 SMC5-9MYC::TRP/ SMC5-9MYC::TRP | This study | |
| CCG1508 | MATa/a leu2/leu2 ura3/ura3 lys2/lys2 trp1/trp1 SMC6-9MYC::TRP / SMC6-9MYC::TRP | | This study |
| CCG1981 | MATa/α ho::LYS2/ho::LYS2 lys2/lys2 ura3/ura3 leu2::hisG/leu2::hisG trp1::hisG/trp1::hisG arg4-nsp/arg4-nsp his4x/his4x smc5-6::NAT/smc5-6::NAT | | This study |
| CCG1985 | MATa/α ho::LYS2/ho::LYS2 lys2/lys2 ura3/ura3 leu2::hisG/leu2::hisG trp1::hisG/trp1::hisG arg4-nsp/arg4-nsp his4x/his4x smc6-9::NAT/smc6-9::NAT | This study | |
| CCG2009 | MATa/α ho::LYS2/ho::LYS2 lys2/lys2 ura3/ura3 leu2::hisG/leu2::hisG trp1::hisG/trp1::hisG arg4-nsp/arg4-nsp his4x/his4x | This study | |
| CCG2132 | MATa/α ho::LYS2/ho::LYS2 lys2/lys2 ura3/ura3 leu2::hisG/leu2::hisG trp1::hisG/trp1::hisG arg4-nsp/arg4-nsp his4x/his4x nse5-ts-1-9MYC::TRP/nse5-ts-1-9MYC::TRP | This study | |
| CCG2396 | MATa/α ho::LYS2/ho::LYS2 lys2/lys2 ura3/ura3 leu2::hisG/leu2::hisG trp1::hisG/trp1::hisG arg4-nsp/arg4-nsp his4x/his4x spo11Δ::KAN/spo11Δ::KAN | | This study |
| CCG2407 | MATa/α ho::LYS2/ho::LYS2 lys2/lys2 ura3/ura3 leu2::hisG/leu2::hisG trp1::hisG/trp1::hisG arg4-nsp/arg4-nsp his4x/his4x nse3-ts12-9MYC::TRP/nse3-ts12-9MYC::TRP | This study | |
| CCG2422 | *MATa/α ho::LYS2/ho::LYS2 lys2/lys2 ura3/ura3 leu2::hisG/leu2::hisG trp1::hisG/trp1::hisG arg4-nsp/arg4-nsp his4x/his4x ndt80Δ::KAN/ndt80Δ::KAN SMC6-9MYC::TRP/SMC6-9MYC::TRP* | This study | |
| CCG2424 | *MATa/α ho::LYS2/ho::LYS2 lys2/lys2 ura3/ura3 leu2::hisG/leu2::hisG trp1::hisG/trp1::hisG arg4-nsp/arg4-nsp his4x/his4x pch2Δ::KAN/pch2Δ::KAN smc6-9::NAT/smc6-9::NAT* | This study | |
| CCG2425 | *MATa/α ho::LYS2/ho::LYS2 lys2/lys2 ura3/ura3 leu2::hisG/leu2::hisG trp1::hisG/trp1::hisG arg4-nsp/arg4-nsp his4x/his4x pch2Δ::KAN/pch2Δ::KAN* | This study | |
| CCG2429 | *MATa/α ho::LYS2/ho::LYS2 lys2/lys2 ura3/ura3 leu2::hisG/leu2::hisG trp1::hisG/trp1::hisG arg4-nsp/arg4-nsp his4x/his4x smc6-9::NAT/smc6-9::NAT spo11Δ::KAN/spo11Δ::KAN* | This study | |
| CCG3733 | *MATa/α ho::lys2/ho::lys2 leu2::hisG/leu2::hisG lys2/lys2 ura3/ura3 spo11-Y135-HA::URA3/spo11-Y135-HA::URA3 arg4N/arg4N* | This study | |
| CCG3818 | *MATa/α ho::LYS2/ho::LYS2 lys2/lys2 ura3/ura3 leu2::hisG/leu2::hisG trp1::hisG/trp1::hisG arg4-nsp/arg4-nsp his4x/his4x nse2ΔC::HYG/nse2ΔC::HYG* | This study | |
| CCG3830 | *MATa/α leu2/leu2 ura3/ura3 lys2/lys2 trp1/trp1 SMC6-9MYC::TRP/SMC6-9MYC::TRP spo11Δ::KAN/spo11Δ::KAN* | This study | |
| CCG3970 | *MATa/α ho::hisG/ho::hisG leu2::hisG/leu2::hisG ura3(∆Sma-Pst)/ura3(∆Sma-Pst) HIS4::LEU2-(BamHI; +ori)/his4-X::LEU2-(NgoMIV; +ori)::URA3* | This study | |
| CCG3976 | *MATa/α ho::hisG/ho::hisG leu2::hisG/leu2::hisG ura3(∆Sma-Pst)/ura3(∆Sma-Pst) HIS4::LEU2-(BamHI; +ori)/his4-X::LEU2-(NgoMIV; +ori)::URA3 smc6-9::NAT/smc6-9::NAT* | This study | |
| CCG4498 | *MATa/α ho::lys2/ho::lys2 leu2::hisG/leu2::hisG lys2/lys2 ura3/ura3 spo11-Y135-HA::URA3/spo11-Y135-HA::URA3 arg4N/arg4N smc6-9::NAT/smc6-9::NAT* | This study | |
| CCG4678 | *MATa/α ho::LYS2/ho::LYS2 lys2/lys2 ura3/ura3 leu2::hisG/leu2::hisG trp1::hisG/trp1::hisG arg4-nsp/arg4-nsp his4x/his4x spo11Δ::KAN/spo11Δ::KAN spo13Δ::KAN/spo13Δ::KAN* | This study | |
| CCG4680 | *MATa/α ho::LYS2/ho::LYS2 lys2/lys2 ura3/ura3 leu2::hisG/leu2::hisG trp1::hisG/trp1::hisG arg4-nsp/arg4-nsp his4x/his4x spo11Δ::KAN/spo11Δ::KAN spo13Δ::KAN/spo13Δ::KAN smc6-9::NAT/smc6-9::NAT* | This study | |
| CCG4874 | *MATa/α leu2-3,112/leu2-3,112 his4-260/his4-260 trp1-289/trp1-289 ARG4/ARG4 thr1-4/thr1-4 ura3-1/ura3-1 ade2-1/ade2-1 CYH10/CYH10 smc6-9::NAT/smc6-9::NAT* | This study | |
| CCG5019 | *MATa/α leu2-3,112/leu2-3,112 his4-260/his4-260 trp1-289/trp1-289 ARG4/ARG4 thr1-4/thr1-4 ura3-1/ura3-1 ade2-1/ade2-1 CYH10/CYH10 smc6-9::NAT/smc6-9::NAT* | This study | |
| CCG6585 | *MATa ho::hisG lys2 ura3 leu2::hisG trp1::hisG URA3-CEN15 LEU2-chXV LYS2-chXV smc6-9::NAT / MATα ho::hisG lys2 ura3 leu2::hisG trp1::hisG TRP1-CEN15 smc6-9::NAT* | This study | |
| CCG6840 | *MATa/α ho::LYS2/ho::LYS2 lys2/lys2 ura3/ura3 leu2::hisG/leu2::hisG trp1::hisG/trp1::hisG arg4-nsp/arg4-nsp his4x/his4x mad2Δ::HYG/mad2Δ::HYG* | This study | |
| CCG6844 | *MATa ho::hisG lys2 ura3 leu2::hisG trp1::hisG URA3-CEN15 LEU2-chXV LYS2-chXV / MATα ho::hisG lys2 ura3 leu2::hisG trp1::hisG TRP1-CEN15* | This study | |
| CCG6864 | *MATa/α ho::LYS2/ho::LYS2 lys2/lys2 ura3/ura3 leu2::hisG/leu2::hisG his3::hisG/his3::hisG trp1::hisG/trp1::hisG leu2::pURA3-TetR-GFP::LEU2/leu2::pURA3-TetR-GFP::LEU2 ura3::TETOx224::URA3/ura3::TETOx224::URA3* | This study | |
| CCG6866 | *MATa/α ho::LYS2/ho::LYS2 lys2/lys2 ura3/ura3 leu2::hisG/leu2::hisG trp1::hisG/trp1::hisG arg4-nsp/arg4-nsp his4x/his4x mad2Δ::HYG/mad2Δ::HYG smc6-9::NAT/smc6-9::NAT* | This study | |
| CCG6937 | *MATa/α ho::LYS2/ho::LYS2 lys2/lys2 ura3/ura3 leu2::hisG/leu2::hisG his3::hisG/his3::hisG trp1::hisG/trp1::hisG leu2::pURA3-TetR-GFP::LEU2/leu2::pURA3-TetR-GFP::LEU2 ura3::TETOx224::URA3/ura3::TETOx224::URA3 smc6-9::NAT/smc6-9::NAT* | This study | |
| CCG7178 | *MATa/α ho::LYS2/ho::LYS2 lys2/lys2 ura3/ura3 leu2::hisG/leu2::hisG trp1::hisG/trp1::hisG arg4-nsp/arg4-nsp his4x/his4x rad9Δ::NAT/rad9Δ::NAT smc6-9::NAT/smc6-9::NAT* | This study | |
| CCG7182 | *MATa/α ho::LYS2/ho::LYS2 lys2/lys2 ura3/ura3 leu2::hisG/leu2::hisG trp1::hisG/trp1::hisG arg4-nsp/arg4-nsp his4x/his4x rad9Δ::NAT/rad9Δ::NAT* | This study | |
| BR1919 | *MATa/α leu2-3,112/leu2-3,112 his4-260/his4-260 trp1-289/trp1-289 ARG4/ARG4 thr1-4/thr1-4 ura3-1/ura3-1 ade2-1/ade2-1 CYH10/CYH10* | P. San Segundo | |
| BR1919-2N | *MATa/MATα leu2-3,112/leu2-3,112 his4-260/his4-260 ura3-1/ura3-1 ade2-1/ade2-1 thr1-4/thr1-4 trp1-289/trp1-289* | P. San Segundo | |
| DP428 | *MATa/MATα leu2-3,112/leu2-3,112 his4-260/his4-260 ura3-1/ura3-1 ade2-1/ade2-1 thr1-4/thr1-4 trp1-289/trp1-289 lys2Δ/ lys2Δ zip1::LYS2/zip1::LYS2 ndt80::LEU2/ndt80::LEU2* | P. San Segundo | |
| DP687 | *MATa/MATα leu2-3,112/leu2-3,112 his4-260/his4-260 ura3-1/ura3-1 ade2-1/ade2-1 thr1-4/thr1-4 trp1-289/trp1-289 zip1::kanMX6/zip1::kanMX6 ndt80::LEU2/ndt80::LEU2 smc6-9::natMX4/smc6-9::natMX4* | P. San Segundo | |
| DP727 | *MATa/MATα leu2-3,112/leu2-3,112 his4-260/his4-260 ura3-1/ura3-1 ade2-1/ade2-1 thr1-4/thr1-4 trp1-289/trp1-289 zip1::kanMX6/zip1::kanMX6 ndt80::LEU2/ndt80::LEU2 smc6-9::natMX4/smc6-9::natMX4 spo11::hphMX4/spo11::hphMX4* | P. San Segundo | |
| DP728 | *MATa/MATα leu2-3,112/leu2-3,112 his4-260/his4-260 ura3-1/ura3-1 ade2-1/ade2-1 thr1-4/thr1-4 trp1-289/trp1-289 zip1::kanMX6/zip1::kanMX6 ndt80::LEU2/ndt80::LEU2 spo11::hphMX4/spo11::hphMX4* | P. San Segundo | |
